# Human Oral Isolate *Lactobacillus fermentum* AGR1487 Reduces Intestinal Barrier Integrity by Increasing the Turnover of Microtubules in Caco-2 Cells

**DOI:** 10.1371/journal.pone.0078774

**Published:** 2013-11-14

**Authors:** Rachel C. Anderson, Wayne Young, Stefan Clerens, Adrian L. Cookson, Mark J. McCann, Kelly M. Armstrong, Nicole C. Roy

**Affiliations:** 1 Food Nutrition & Health Team, Food & Bio-based Products Group, AgResearch Grasslands, Palmerston North, New Zealand; 2 Riddet Institute, Massey University, Palmerston North, New Zealand; 3 Proteins & Biomaterials Team, Food & Bio-based Products Group, AgResearch Lincoln, Lincoln, New Zealand; 4 Rumen Microbiology Team, Animal Health & Nutrition Group, AgResearch Grasslands, Palmerston North, New Zealand; 5 Gravida: National Centre for Growth and Development, The University of Auckland, Auckland, New Zealand; Institut Pasteur de Lille, France

## Abstract

*Lactobacillus fermentum* is found in fermented foods and thought to be harmless. *In vivo* and clinical studies indicate that some *L. fermentum* strains have beneficial properties, particularly for gastrointestinal health. However, *L. fermentum* AGR1487 decreases trans-epithelial electrical resistance (TEER), a measure of intestinal barrier integrity. The hypothesis was that *L. fermentum* AGR1487 decreases the expression of intestinal cell tight junction genes and proteins, thereby reducing barrier integrity. Transcriptomic and proteomic analyses of Caco-2 cells (model of human intestinal epithelial cells) treated with *L. fermentum* AGR1487 were used to obtain a global view of the effect of the bacterium on intestinal epithelial cells. Specific functional characteristics by which *L. fermentum* AGR1487 reduces intestinal barrier integrity were examined using confocal microscopy, cell cycle progression and adherence bioassays. The effects of TEER-enhancing *L. fermentum* AGR1485 were investigated for comparison. *L. fermentum* AGR1487 did not alter the expression of Caco-2 cell tight junction genes (compared to *L. fermentum* AGR1485) and tight junction proteins were not able to be detected. However, *L. fermentum* AGR1487 increased the expression levels of seven tubulin genes and the abundance of three microtubule-associated proteins, which have been linked to tight junction disassembly. Additionally, Caco-2 cells treated with *L. fermentum* AGR1487 did not have defined and uniform borders of zona occludens 2 around each cell, unlike control or AGR1485 treated cells. *L. fermentum* AGR1487 cells were required for the negative effect on barrier integrity (bacterial supernatant did not cause a decrease in TEER), suggesting that a physical interaction may be necessary. Increased adherence of *L. fermentum* AGR1487 to Caco-2 cells (compared to *L. fermentum* AGR1485) was likely to facilitate this cell-to-cell interaction. These findings illustrate that bacterial strains of the same species can cause contrasting host responses and suggest that food-safe status should be given to individual strains not species.

## Introduction

It is thought that symbiotic bacteria of the human gastrointestinal tract may be harmful under certain conditions [1]. The term ‘pathobionts’ is used to describe microbes with pathogenic potential associated with chronic inflammatory conditions linked to environmental and/or genetic alterations [2]. Pathobionts differ from opportunistic pathogens (which often cause acute infections and are typically acquired from the environment) because they are harmless to a healthy host.

*Lactobacillus fermentum* is commonly found in fermented food products and is considered a “generally recognised as safe” (GRAS) organism by the US Food and Drug Administration (FDA). *In vivo* studies indicate that some *L. fermentum* strains have beneficial properties, particularly in relation to gastrointestinal health. For example, *L. fermentum* BR11 reduces colitis in rats [3]–[5], *L. fermentum* CECT5716 prevents and alleviates intestinal damage induced in mice [6], and *L. fermentum* ACA-DC 179 protects against colitis and *Salmonella* infection induced in mice [7]. In addition, clinical studies have also shown the benefits of some *L. fermentum* strains. In particular, *L. fermentum* CECT5716 enhances the effect of the influenza vaccination in healthy adults [8], reduces re-infection in women with infectious mastitis [9], and reduces the incidence of upper respiratory and gastrointestinal tract infections in six month old infants [10].

In contrast to these beneficial properties, our previous research indicated that some strains of *L. fermentum* may have a negative effect on intestinal barrier integrity. In that research, of the five *L. fermentum* human oral isolates studied, three strains reduced the trans-epithelial electrical resistance (TEER) across Caco-2 cell layers (indicating they had a negative effect on the integrity of the tight junctions between the adjacent epithelial cells), one *L. fermentum* strain increased TEER compared to control media (indicating that it enhanced barrier integrity) and the other did not alter TEER compared to control media [11]. The strain that caused the largest decrease in TEER compared to control media was *L. fermentum* AGR1487 and the strain that caused an increase in TEER compared to control media was *L. fermentum* AGR1485. These results suggested that some *L. fermentum* strains exhibit detrimental effects during targeted *in vitro* studies and that further research was warranted to understand the mechanisms involved.

Bacteria are known to alter intestinal barrier integrity using a variety of mechanisms [12]–[14]. For example, some enteropathogenic *Escherichia coli* (EPEC) strains reduce tight junction integrity by attaching to epithelial cells and causing cytoskeleton rearrangement [15]. In contrast, *Lactobacillus* species can enhance intestinal barrier integrity in many different ways including increasing the expression levels of genes and abundance of proteins involved in the formation of tight junctions that seal the space between adjacent epithelial cells [11], [16]–[19], increasing the transcription of the genes encoding the adherence junction E-Cadherin/β-Catenin protein complex [20], and activating p38 and ERK signalling [21].

The hypothesis of this research was that *L. fermentum* AGR1487 decreases the expression levels of intestinal epithelial cell tight junction genes and proteins, thereby reducing barrier integrity. Transcriptomic and proteomic analyses of Caco-2 cells (a model of human intestinal epithelial cells) treated with *L. fermentum* AGR1487 were used to obtain a global view of the effect of the bacterium on intestinal epithelial cells. Specific functional characteristics by which *L. fermentum* AGR1487 reduces intestinal barrier integrity were examined using confocal microscopy, cell cycle progression and adherence bioassays. The effects of TEER-enhancing *L. fermentum* AGR1485 were also investigated for comparison.

## Materials and Methods

### Ethics statement

The two bacterial strains used in this research were isolated from saliva swabs of adult human volunteers as previously described [11]. Ethical approval from the New Zealand Health and Disability Committee was not required under Section 3 of the *Standard Operating Procedures of the Health and Disability Committee* due to the non-invasive nature of the sampling and the healthy status of the volunteers. Written consent for collection and use of the samples for research purposes was obtained from the volunteers. *L. fermentum* AGR1485 was isolated from a healthy individual and *L. fermentum* AGR1487 was isolated from a then healthy individual who was later diagnosed with inflammatory bowel disease (IBD).

### Bacterial strains and mammalian cell culture

The bacteria were grown in MRS broth (de Man, Rogosa and Sharpe Broth; Difco) overnight in 5% CO_2_ at 37°C. Caco-2 cell (ATCC HTB-37) stock cultures were grown in T75 flasks in M199 with 10% foetal bovine serum (GIBCO, Invitrogen Corporation), 1% non-essential amino acids (MEM non-essential amino acids 100× solution; Sigma-Aldrich) and 1% penicillin-streptomycin (10000 units penicillin G sodium salt and 10000 µg streptomycin sulphate in 0.85% saline; GIBCO, Invitrogen Corporation) at 37°C in 5% CO_2_. The media was replaced every 3–4 days and the cells were subcultured weekly at a ratio of 1∶3.

### TEER assay

The TEER assays were carried out using confluent undifferentiated Caco-2 cells (5 days old; Passage number between 30 and 35) grown on 14 mm collagen membrane inserts (Cellagen Discs CD-24; MP Biomedicals) as previously described [11], [22]. It was sufficient to use monolayers of this age because at this stage in development the tight junctions between the cells have already formed, and a previous study showed that the TEER response of differentiated versus undifferentiated Caco-2 monolayers was comparable [11], [22]. The bacteria were prepared by growing overnight cultures (MRS broth, 37°C, 5% CO_2_), collecting the bacterial cells by centrifugation (20,000×*g* for 5 minutes) and suspending the pellet in M199 with 1% non-essential amino acids to an optical density at 600 nm (OD_600 nm_) of 0.9, unless otherwise stated. The bacterial supernatants were prepared by incubating the bacteria at OD_600 nm_ of 0.9 in M199 with 1% non-essential amino acids overnight in 5% CO_2_ at 37°C, then removing the bacterial cells by centrifugation (20,000×*g* for 5 min) and filter sterilisation (Millex GP 0.22 µm syringe driven filter; Millipore). Each treatment was tested in quadruplicate per assay and each assay was repeated three times. Treatments were compared in GenStat v11.1 using residual maximum likelihood (REML) analysis with an unstructured covariance model to take account of the repeated measures.

### ^3^H-mannitol flux assay

Caco-2 monolayers were grown on collagen membrane inserts as described for the TEER assays. The cells were prepared the day before the assay by removing the media, washing three times with 0.1M phosphate buffered saline (PBS, pH 7.4) and adding M199 with 1% non-essential amino acids. The bacterial solutions were prepared as described for the TEER assay and 2.5 µCi/mL of ^3^H-mannitol was added to each 250 µL treatment solution. Each treatment was tested in quadruplicate. Every two hours, 100 µL samples of the basal media were removed. The samples were mixed with 2 mL Starscint Scintillation Fluid and the ^3^H-mannitol was measured using a Luminescence counter (PerkinElmer 1450 MicroBeta TriLux). The percentage of ^3^H-mannitol that crossed the cell layer was calculated and plotted against time. Treatments were compared in GenStat v11.1 using REML analysis.

### Gene and protein expression analysis

The aim of the gene and protein expression study was to investigate the expression of all biological pathways that may directly or indirectly effect barrier integrity (not just the tight junction genes and proteins); therefore, it was necessary for the cells to be differentiated into mature epithelial cells for the data to be relevant. Caco-2 cells were grown in T75 flasks for 18 days to allow them to differentiate in polarised epithelial cells. The media was replaced every 3–4 days. The bacterial solutions were prepared as described for the TEER assay. The media was removed from the Caco-2 monolayers and replaced with the treatment solution: 1) cell culture media (M199 with 1% non-essential amino acids); 2) *L. fermentum* AGR1485 suspended in cell culture media; and 3) *L. fermentum* AGR1487 suspended in cell culture media. There were eight replicates per treatment group. After 8 hours of exposure (37°C, 5% CO_2_) the treatment solutions were removed and the monolayers were rinsed with PBS. The total RNA and protein were extracted from the samples using the TRIzol method, and gene expression and protein abundance were quantified using whole genome microarray and LC-MS/MS analyses respectively as described in Text S1. The microarray data was validated by real-time PCR using the method also described in Text S1. The ten genes chosen for validation had a range of expression patterns across the microarray analysis and are involved in a range of biological processes.

### Confocal microscopy

Caco-2 cells were grown on Permanox™ coated slides (In Vitro Technologies) for seven days until confluent. Caco-2 cells were treated with M199 media (control), M199 media containing OD_600 nm_ 0.9 *L. fermentum* AGR1485 or M199 media containing OD_600 nm_ 0.9 *L. fermentum* AGR1487 at 37°C with 5% CO_2_ for 8 hours. There were four replicates per treatment group. Following treatment, the Caco-2 cells were rinsed with PBS three times (added dropwise), fixed in 4% (w/v) paraformaldehyde for 20 minutes, quenched/permeabilised with 50 mM NH_4_Cl (in PBS) for 15 minutes and blocked with blocking buffer (2% (v/v) foetal bovine serum, 1% SSA, 0.1% Triton X-100, 0.05% Tween 20 in PBS, pH 7.2) for 20 minutes. The Caco-2 cells were then immuno-stained with the primary antibody, rabbit anti- zona occludens 2 (ZO-2; also called tight junction protein 2 (TJP2)) (1∶100), in blocking buffer for 1 hour, followed by a PBS wash for 10 minutes to reduce non-specific staining (0.1% Triton X-100, 0.05% Tween 20 in PBS), and the secondary antibody, Alexa Fluor 488 goat anti-rabbit IgG (1∶200), in blocking buffer for 1 hour. The slides were imaged with a confocal microscope (Leica TCS SP5) with the Argon laser excitation emissions between 500 and 540 nm. The images were viewed using LAS AF Lite (Leica Application Suite) v1.8.2 software.

### Cell cycle analysis

To assess the effect of the bacterial strains on Caco-2 cell cycle progression, the Caco-2 cells were grown in T75 flasks and treated with the bacterial strains as described for the whole genome and proteome analysis. DNA content analysis was based on the incorporation of propidium iodide into DNA and the resulting fluorescence a measure of the relative proportion of cells in the various stages of the cell cycle [23]. Six replicates of each treatment groups were assessed. Each sample was prepared and analysed using a FACSCalibur flow cytometer (Becton Dickinson, NZ) as previously described [24], [25]. The fluorescence intensity of propidium iodide was collected at 585 nm, using CELLQuest Pro Software (Becton Dickinson) and analysed for DNA content using FlowJo (TreeStar V7.6.3). Treatments were compared in GenStat v11.1 using ANOVA.

### Cell adherence

To assess bacterial adherence to epithelial cells, Caco-2 cells were grown in 24-well plates 37°C with 5% CO_2_ for 18 days until differentiated. Caco-2 monolayers were prepared for the experiment by removing the media, washing three times with PBS and adding M199 with 1% non-essential amino acids (without foetal bovine serum and penicillin-streptomycin). *L. fermentum* AGR1485 and ARG1487 were grown overnight in MRS broth and approximately 10^7^ (10 µL) were added to each well with each strain being assessed in triplicate. After 3 hours incubation at 37°C in 5% CO_2_, bacteria were removed from one 24-well tissue culture plate and the Caco-2 cells were washed gently five times with pre-warmed PBS. Bacterial separation from Caco-2 cells was mediated by adding 1 mL of a 1% solution of Triton X-100 to each well and stirring using a stir bar and magnetic plate for 10 minutes. Bacteria were quantified by using 20 µL dilution spots on Luria Bertani (LB) agar plates. To assess adherence over 6 hours, non-adherent bacteria were removed after 3 hours and the epithelial cells were washed gently five times with PBS as described previously. Fresh media was then added to each well and the tissue culture plate incubated for a further 3 hours prior to enumeration. Treatments were compared in GenStat v11.1 using ANOVA.

## Results

### *L. fermentum* AGR1487 had a negative effect on *in vitro* intestinal barrier integrity

**Figure 1:**
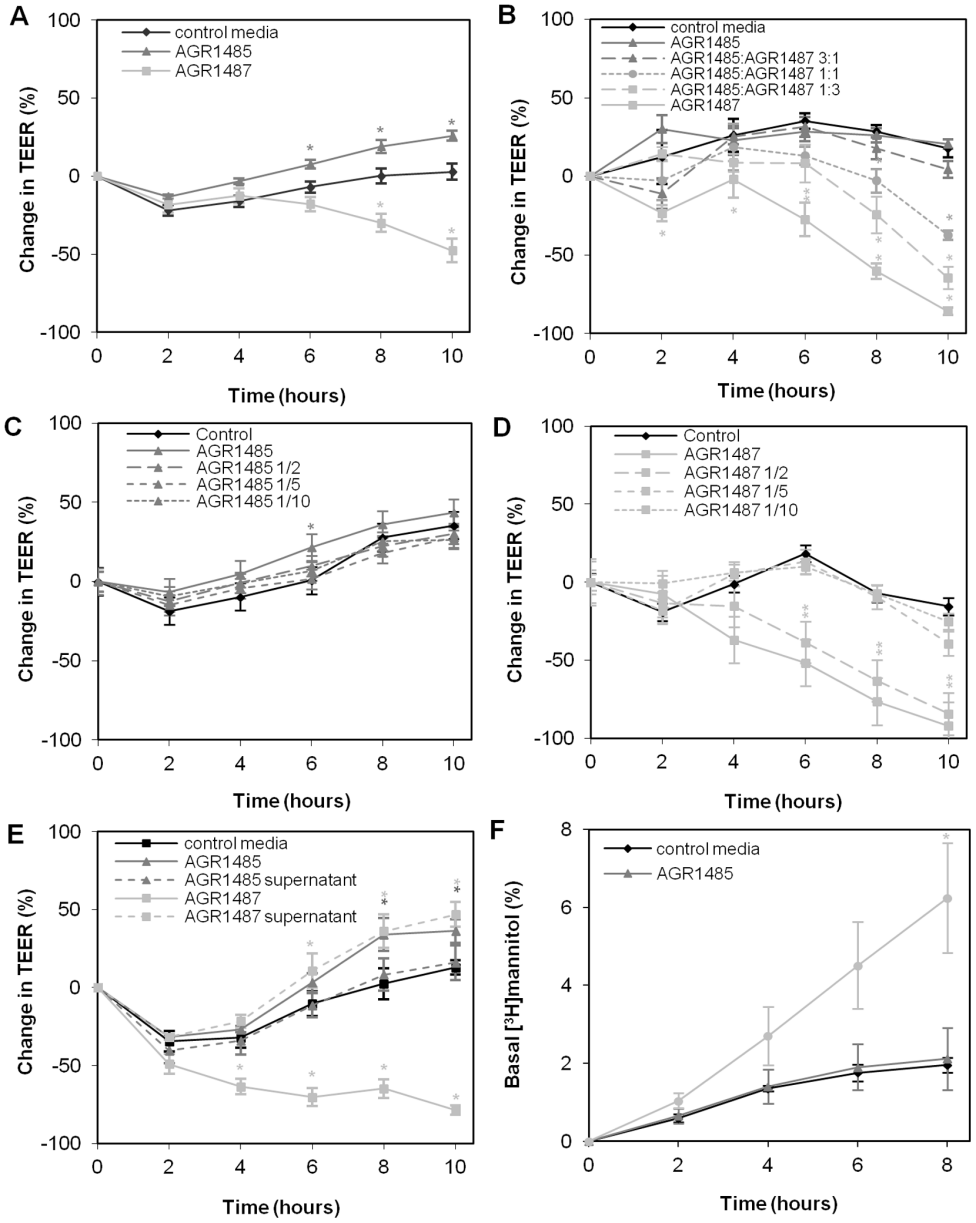
Effect of *L. fermentum* AGR1485 and *L. fermentum* AGR1487 on *in vitro* measures of intestinal barrier function. **A–E** Change in trans-epithelial electrical resistance (TEER) across Caco-2 cell monolayers over time in the presence of: **A** *L. fermentum* AGR1485 and *L. fermentum* AGR147; **B** *L. fermentum* AGR1485 and *L. fermentum* AGR1487 mixed at different ratios; **C** Different concentrations of *L. fermentum* AGR1485 (OD_600 nm_ 0.9 diluted to 1/2, 1/5 and 1/10); **D** Different concentrations of *L. fermentum* AGR1487 (OD_600 nm_ 0.9 diluted to 1/2, 1/5 and 1/10); **E** *L. fermentum* AGR1485 and *L. fermentum* AGR1487 supernatants. **F** ^3^H-mannitol flux across Caco-2 cell monolayers in the presence of *L. fermentum* AGR1485 and *L. fermentum* AGR1487. The values plotted are the means for four monolayers and the error bars show the SEM. * P<0.05 compared to control media.

The TEER assay was used to measure the effects of *L. fermentum* AGR1485 and AGR1487 on paracellular ion permeability, a measure of the integrity of tight junctions between Caco-2 cells. *L. fermentum* AGR1487 had a negative effect on tight junction integrity in all assays and reduced TEER by between 51% and 92% compared to control media after 10 hours (Figures 1a, 1b, 1d and 1e). The effect of *L. fermentum* AGR1485 was inconsistent; it had a positive effect on TEER compared to control media in some assays (Figures 1a and 1e); in the other assays *L. fermentum* AGR1485 did not cause a change in TEER compared to control media (Figures 1b and 1c). When *L. fermentum* AGR1485 and *L. fermentum* AGR1487 were added together to Caco-2 cells, the negative effect of *L. fermentum* AGR1487 overcame the positive/neutral effect of *L. fermentum* AGR1485 (Figure 1b); at a ratio of 1∶1 the TEER decreased by 56% after 10 hours. The TEER-reducing effect of *L. fermentum* AGR1487 was dose dependent (Figure 1d). The negative effects of *L. fermentum* AGR1487 on TEER remained when half the number of bacteria cells was added, but one fifth and one tenth of the number of *L. fermentum* AGR1487 cells did not reduce TEER compared to control media. Although *L. fermentum* AGR1487 has a negative effect on TEER, *L. fermentum* AGR1487 supernatant caused a 34% increase in TEER compared to control media after 10 hours; whereas, *L. fermentum* AGR1485 supernatant did not cause a change in TEER compared to control media (Figure 1e).

As a second measure of tight junction integrity, small molecule paracellular permeability was investigated by monitoring the flux of ^3^H-mannitol across Caco-2 cell monolayers from the apical compartment to the basal compartment. *L. fermentum* AGR1485 did not alter the flux of mannitol across the cell layer compared to control media, but *L. fermentum* AGR1487 caused an increase in mannitol flux across the Caco-2 cell layers, indicating it increased permeability (Figure 1f).

### *L. fermentum* AGR1487 had a larger effect on Caco-2 cell gene expression levels than *L. fermentum* AGR1485

**Figure 2:**
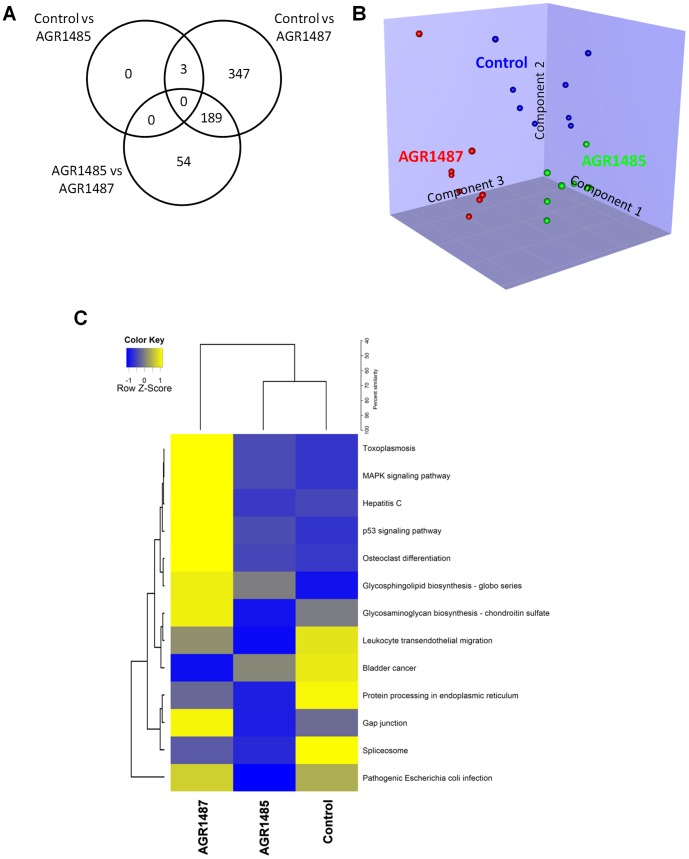
Summary of the gene expression profiles of Caco-2 cells untreated or treated with *L. fermentum* AGR1485 and *L. fermentum* AGR1487 for 8 hours. **A** Venn diagram showing the number of differentially expressed genes in each comparison. **B** Graph showing the Partial Least Squares Discriminant Analysis (PLS-DA) of the gene expression data. The dots represent the individual samples in each treatment group. **C** Heatmap showing the average expression levels of genes within KEGG pathways (indicated by row labels) significantly over-represented among differentially expressed genes between the *L. fermentum* AGR1485 and *L. fermentum* AGR1487 treated Caco-2 cells.

Whole genome expression analysis was used to determine the effect of *L. fermentum* AGR1485 and AGR1487 on Caco-2 cell gene expression. The raw microarray data have been deposited in the NCBI Gene Expression Omnibus under the GEO accession number GSE46352. As shown in the Venn diagram (Figure 2a), compared to control media, *L. fermentum* AGR1485 altered the expression levels of three genes (Table S1), whereas, *L. fermentum* AGR1487 altered the expression levels of 539 genes (Table S2). Partial least squares discriminant analysis (PLS-DA) showed that the three treatment groups could be discriminated based on their gene expression profiles (Figure 2b).

Genes that were differentially expressed in Caco-2 cells treated with *L. fermentum* AGR1487 compared to *L. fermentum* AGR1485 were significantly over-represented in a number of KEGG pathways, the top 5 of which were *Pathogenic Escherichia coli infection*, *Gap junction*, *Glycosaminoglycan biosynthesis - chondroitin sulphate*, *MAPK signaling pathway* and *Phagosome* (Table 1). Hierarchical clustering of average expression levels of genes within these significantly over-represented KEGG pathways showed that the Caco-2 cells exposed to *L. fermentum* AGR1485 were more similar to that of the control Caco-2 cells than those exposed to *L. fermentum* AGR1487 (Figure 2c).

**Table 1:** KEGG pathways containing an enrichment of differentially expressed genes in Caco-2 cells treated with with *L. fermentum* AGR1485 compared to *L. fermentum* AGR1487 for 8 hours.

KEGG ID	P value	Odds Ratio	Exp Count	Count	Size	KEGG Pathway	Genes
Hsa05130	0.0000	12.42	0.81	8	56	Pathogenic Escherichia coli infection	TUBA4A, EZR, TUBA1B, TUBB3, TUBB4, TUBA8, TUBA3D, TUBB8
Hsa04540	0.0003	6.17	1.30	7	90	Gap junction	TUBA4A, TUBA1B, TUBB3, TUBB4, TUBA8, TUBA3D, TUBB8
Hsa00532	0.0037	11.10	0.32	3	22	Glycosaminoglycan biosynthesis - chondroitin sulfate	CHPF2, XYLT2, B3GALT6
Hsa04010	0.0050	2.86	3.88	10	268	MAPK signalling pathway	DUSP1, DUSP4, FOS, NR4A1, HSPA1A, HSPA6, HSPB1, JUN, GADD45B, GADD45G
Hsa04145	0.0064	3.47	2.22	7	153	Phagosome	TUBA4A, TUBA1B, TUBB3, TUBB4, TUBA8, TUBA3D, TUBB8
Hsa04141	0.0096	3.20	2.39	7	165	Protein processing in endoplasmic reticulum	HSPA1A, HSPA6, HSP90AA1, DNAJB1, PPP1R15A, SAR1A, MAN1C1
Hsa04380	0.0102	3.53	1.85	6	128	Osteoclast differentiation	FOS, FOSB, JUN, FOSL1, SOCS1, SOCS3
Hsa04115	0.0165	4.42	0.98	4	68	p53 signaling pathway	CDKN1A, SFN, GADD45B, GADD45G
Hsa00603	0.0168	11.59	0.20	2	14	Glycosphingolipid biosynthesis - globo series	FUT1, GLA
Hsa05219	0.0224	5.39	0.61	3	42	Bladder cancer	CDKN1A, MMP1, RASSF1
Hsa04670	0.0259	3.20	1.68	5	116	Leukocyte transendothelial migration	CLDN4, CLDN3, VASP, EZR, BCAR1
Hsa03040	0.0363	2.90	1.84	5	127	Spliceosome	HSPA1A, HSPA6, SRSF6, SRSF7, RBM8A
Hsa05145	0.0418	2.78	1.91	5	132	Toxoplasmosis	HSPA1A, HSPA6, IL10RA, NOS2, SOCS1
Hsa05160	0.0441	2.74	1.94	5	134	Hepatitis C	CDKN1A, CLDN4, CLDN3, SOCS3, TICAM1

To validate the microarray data, the expression levels of ten genes were also quantified using real-time PCR (qPCR) and the results were compared with the gene expression data obtained using microarray analysis (Table S3). For the main comparison of interest, Caco-2 cells treated with *L. fermentum* AGR1487 versus untreated Caco-2 cells, there was a significant correlation between the qPCR and microarray results (Pearson coefficient = 0.68; P = 0.04), supporting the validity of the microarray data.

### *L. fermentum* AGR1487 did not alter the expression levels of Caco-2 cell genes in the tight junction pathway but altered other tight junction related genes

Analysis of the KEGG pathways showed differentially expressed genes between the Caco-2 cells treated with *L. fermentum* AGR1485 compared to *L. fermentum* AGR1487 were not over-represented in the *Tight Junction* pathway (P = 0.57). However, 7 tubulin genes (TUBA4A, TUBA1B, TUBB3, TUBB4, TUBA8, TUBA3D, TUBB8) had increased expression levels in response to *L. fermentum* AGR1487 compared to *L. fermentum* AGR1485 (fold-change 1.6 to 2.8; FDR<0.05). These tubulin genes are present in the *Gap Junction* and *Pathogenic Escherichia coli Infection* KEGG pathways (Table 1).

**Figure 3:**
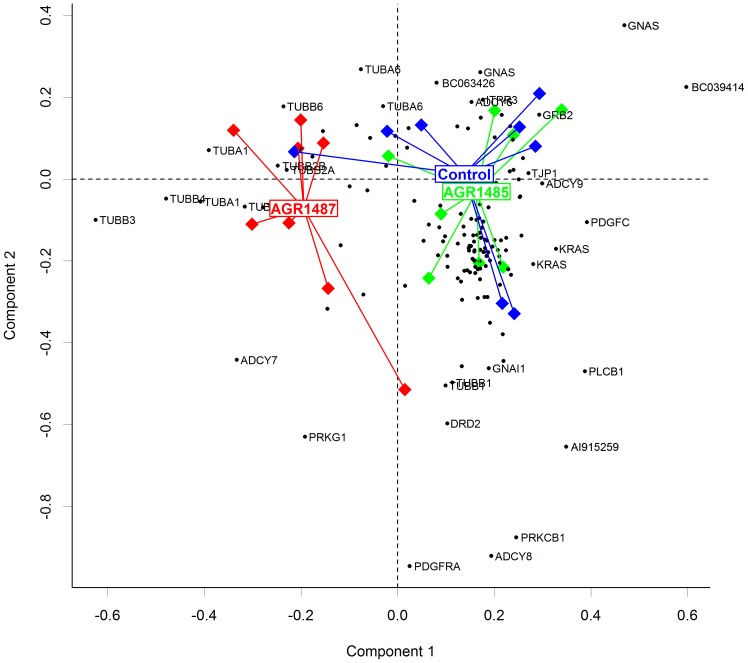
Correspondence analysis (CA) of the expression of genes involved in the KEGG *Gap Junction* pathway in Caco-2 cells untreated or treated with *L. fermentum* AGR1485 and *L. fermentum* AGR1487 for 8 hours. CA plot shows expression profiles of Caco-2 cells treated with *L. fermentum* AGR1487 clustered separately from untreated cells or those treated with *L. fermentum* AGR1485. Lines lead from individual samples to the centroid of each treatment group. Genes (black points) within the KEGG gap junction pathway are plotted onto the same graph. Genes that show higher contributions to separation of samples are plotted further from origin, and direction from origin indicates closeness of expression pattern with nearby sample. For example, the separation of samples from the AGR1487 treatment group from other treatment groups is largely determined by their expression of the tubulin genes TUBB3, TUBB4, and TUBA1.

Correspondence analysis (CA) of expression levels of genes within the *Gap Junction* pathway showed that expression profiles of Caco-2 cells treated with *L. fermentum* AGR1487 were clearly different from expression profiles of untreated cells or those treated with *L. fermentum* AGR1485 (Figure 3). Projection of *Gap Junction* pathway genes showed that the expression pattern of tubulin genes was primarily what differentiated the *L. fermentum* AGR1487 treated samples from those untreated or treated with *L. fermentum* AGR1485 (Figure 3).

### *L. fermentum* AGR1487 had less effect on Caco-2 cell protein abundance than *L. fermentum* AGR1485

**Figure 4:**
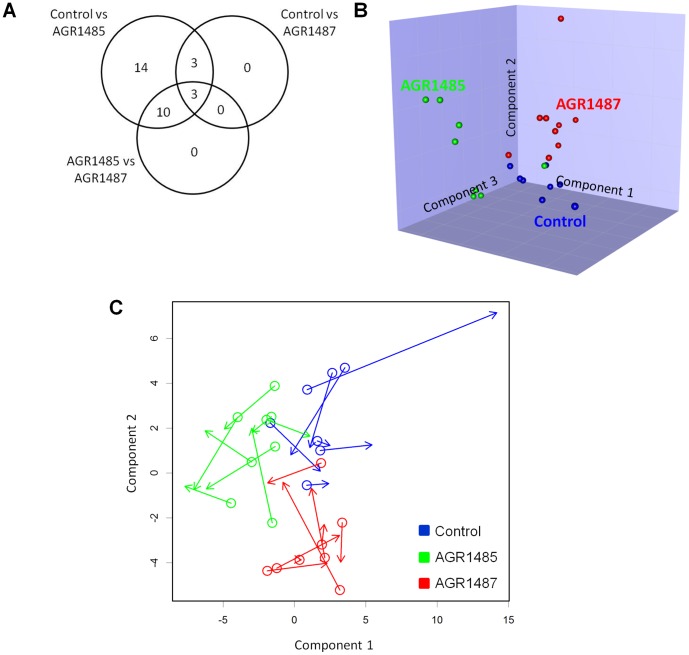
Summary of the protein expression of Caco-2 cells untreated or treated with *L. fermentum* AGR1485 and *L. fermentum* AGR1487 for 8 hours. **A** Venn diagram showing the number of differentially expressed proteins in each comparison. **B** Graph showing the Partial Least Squares Discriminant Analysis (PLS-DA) of the protein expression data. The dots represent the individual samples in each treatment group. **C** Procrustes rotation analysis of gene and protein expression profiles. Open circles indicate PLS-DA projections of gene expression and arrow heads indicate PLS-DA projection of protein expression. This plot indicates that if the protein expression profile of a sample was dissimilar to other samples, then its gene expression profile also tended to be relatively dissimilar.

Caco-2 cell whole protein digests were isobarically labelled and analysed using LC-MS/MS to determine the effect of *L. fermentum* AGR1485 and AGR1487 on protein abundance profiles. Of the 63 proteins detected (abundance significantly higher than background), 30 were differentially expressed in at least one comparison (Table 2). Compared to control media, *L. fermentum* AGR1485 altered the abundance of 30 proteins, whereas *L. fermentum* AGR1487 altered the abundance of only six proteins (Figure 4a). As with the gene expression data, the PLS-DA plot showed that the three treatment groups could also be discriminated based on their protein expression profiles (Figure 4b). Procrustes rotation analysis showed there was a significant correlation (correlation = 0.5; P = 0.0036) between protein and gene expression profiles (Figure 4c).

**Table 2:** Proteins differentially expressed between untreated Caco-2 cells and those treated with with *L. fermentum* AGR1485 and *L. fermentum* AGR1487 for 8 hours.

GI number	Protein name	P value	Control vs AGR1485 fold change	Control vs AGR1487 fold change	AGR1485 vs AGR1487 fold change
47519616	tropomyosin 2 (beta)	0.0000	−2.08*	3.12*	6.56*
221042310	tropomyosin 1 (alpha)	0.0000	−2.17*	3.12*	6.77*
45768281	Histone cluster 1, H3i	0.0002	−2.56*	1.07	2.76*
4506901	serine/arginine-rich splicing factor 3	0.0003	−2.94*	−1.32	2.28*
28373916	Chain A, Crystal Structure Of Human Saposin B	0.0004	−3.03*	−1.15	2.61*
825683	Thymosin, beta 4, Y-linked	0.0005	−4.00*	−1.30	3.08*
119581587	basigin (Ok blood group), isoform CRA_d	0.0007	−4.35*	−1.69*	2.55*
4507761	ubiquitin-60S ribosomal protein L40 precursor	0.0011	−3.23*	−1.43	2.30*
4507949	14-3-3 protein beta/alpha	0.0019	−2.44*	−1.16	2.08*
62898141	prosaposin (variant Gaucher disease and variant metachromatic leukodystrophy) variant	0.0020	−3.03*	−1.09	2.79*
306549	homology to rat ribosomal protein L23, partial	0.0048	−1.54*	1.00	1.53*
10800130	histone H2A type 1-D	0.0076	−3.70*	−1.85*	2.02
744518	FKBP-rapamycin-associated protein	0.0081	−5.56*	−1.72	3.23
10800138	histone H2B type 1-D	0.0127	−3.70*	−2.94	1.25
18105048	histone H2B type 1-K	0.0130	−9.09*	−2.56	3.50
356168	histone H1b	0.0141	−4.76*	−1.54*	3.14
4885377	histone H1.3	0.0145	−5.00*	−1.56*	3.18
1617118	TSA	0.0150	−5.56*	−1.35	3.98
16307067	SUB1 homolog (S. cerevisiae)	0.0153	−1.69*	−1.23	1.36
189054180	cytokeratin 18 (424 AA)	0.0194	−1.67*	−1.37	1.22
38260014	MBP-2 (MHC Binding Protein-2)	0.0275	−4.35*	−1.64	2.70
1477646	plectin	0.0290	−2.17*	1.13	2.45*
7106439	tubulin beta-5 chain	0.0312	−1.59*	−1.19	1.33
4504251	histone H2A type 2-A	0.0331	−1.45*	−1.05	1.37*
223480	dismutase,Cu/Zn superoxide	0.0357	−1.92*	−1.35	1.41
197725012	Chain B, Crystal Structure Of Human Amsh-Lp Dub Domain In Complex With Lys63-Linked Ubiquitin Dimer	0.0369	−1.75*	−1.30	1.36
4504253	histone H2A.x	0.0431	−1.96*	−1.64	1.20
119619135	hCG1790904, isoform CRA_a	0.0440	−1.75*	−1.33	1.30
20664042	Chain A, Crystal Structure Of Calcium-Free (Or Apo) Human S100a6	0.0482	−1.49*	−1.12	1.33
74709215	Putative histone H2B type 2-C	0.0499	−2.38*	−1.12	2.14

* P<0.05 for that comparison.

### *L. fermentum* AGR1487 increased the expression levels of microtubule associated proteins and altered the localisation of zona occludens 2 in Caco-2 cells

Three tubulin proteins were detected in the analysis (tubulin beta-5 chain, tubulin beta and tubulin beta-4), but only one of these had altered abundance in one comparison: the tubulin beta-5 chain protein had reduced abundance in response to *L. fermentum* AGR1485 compared to control cells (fold change = 1.59). However, compared to Caco-2 cells treated with *L. fermentum* AGR1485, Caco-2 cells treated with *L. fermentum* AGR1487 had increased abundance of the tropomyosin 1 (alpha) and tropomyosin 2 (beta) proteins, which are involved in the formation of microtubule bundles, and the plectin protein, which acts as a link between the microtubules and the actin cytoskeleton.

**Figure 5:**
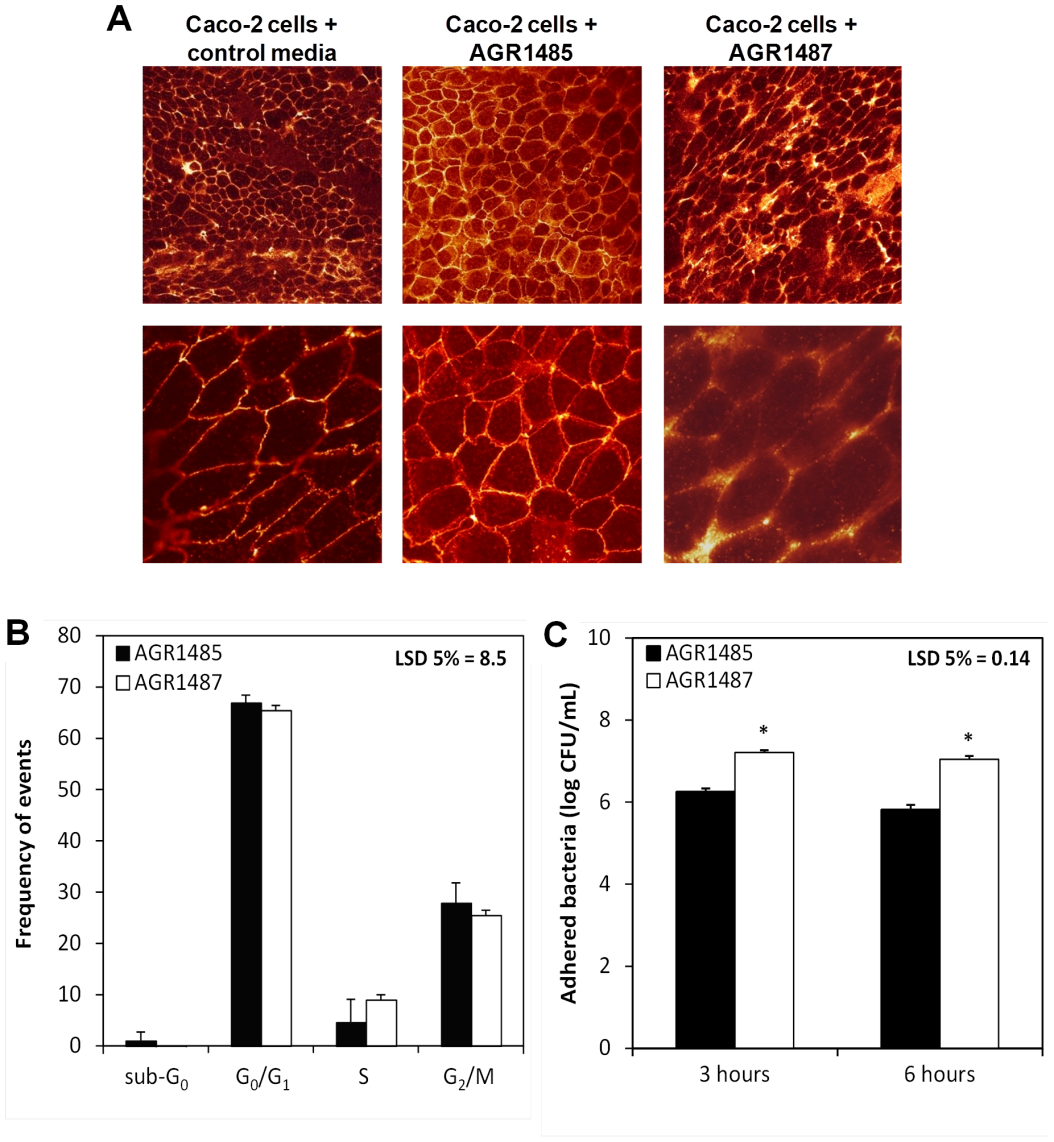
Comparison of the effects of *L. fermentum* AGR1485 and *L. fermentum* AGR1487 on Caco-2 cell tight junction morphology, cell cycle progression and adherence. **A** Confocal microscopy images of immuno-stained zona occludens 2 (ZO-2) of Caco-2 cells untreated or treated with *L. fermentum* AGR1485 and *L. fermentum* AGR1487 for 8 hours. Treatments were completed in quadruplicate and the images shown are representative. **B** Effect of *L. fermentum* AGR1485 and *L. fermentum* AGR1487 on Caco-2 cell cycle phase after treatment for 8 hours. The values plotted are means of six replicates and the error bars show the SEM. There were no significant differences between bacterial treatments. **C** Ability of *L. fermentum* AGR1485 *and L. fermentum* AGR1487 to adherence to Caco-2 cells over time. The values plotted are means of six replicates and the error bars show the SEM. * P<0.05 between bacterial treatments.

No tight junction-related proteins were detected in the analysis so the effect of *L. fermentum* AGR1485 and *L. fermentum* AGR1487 on the Caco-2 cell abundance of these proteins could not be determined. Therefore, confocal microscopy was used to investigate the localisation of the tight junction proteins in Caco-2 cells when treated with the bacteria. Visualisation of the abundance and localisation of ZO-2 showed that Caco-2 cells treated with *L. fermentum* AGR1487 did not have defined and uniform borders of ZO-2 around each cell, unlike control or AGR1485 treated cells. ZO-2 in Caco-2 cells treated with *L. fermentum* AGR1487 was localised in clusters with an uneven distribution (Figure 5a).

### *L. fermentum* AGR1487 did not alter Caco-2 cell cycle progression

*L. fermentum* AGR1487 caused a large decrease in TEER and altered the visual appearance of Caco-2 cells, so DNA content analysis was used to assess whether the bacterium affected Caco-2 cell cycle progression. There were no differences in the number of Caco-2 cells in any cell cycle phase between those treated with *L. fermentum* AGR1485 and AGR1487 (Figure 5b).

### *L. fermentum* AGR1487 adhered to Caco-2 cells in greater numbers than *L. fermentum* AGR1485

*L. fermentum* AGR1487 supernatant did not cause a decrease in TEER, unlike what was observed when Caco-2 cells were treated with the live bacterium, so it was hypothesised that physical interaction between the bacterium and Caco-2 cells was required for the negative effect. The ability for *L. fermentum* AGR1485 and AGR1487 to adhere to Caco-2 cells was determined. Compared to *L. fermentum* AGR1485, the number of *L. fermentum* AGR1487 cells adhered to Caco-2 cells after 3 and 6 hours was 9-fold and 15-fold higher, respectively (Figure 5c).

## Discussion

The results showed that, despite being from a species considered harmless, human oral isolate *L. fermentum* AGR1487 increased the expression levels of genes and abundance of proteins that have been implicated in reducing the integrity of the tight junctions between intestinal epithelial cells. For example, compared to control Caco-2 cells and those treated with *L. fermentum* AGR1485, Caco-2 cells treated with *L. fermentum* AGR1487 had both a higher expression level of genes encoding for tubulins and a higher abundance of microtubule-associated proteins. A high turnover in tubulin synthesis has been linked to the disassembly of tight junctions [26], which indicates that these genes and proteins may play a role in the negative effects on barrier integrity observed for *L. fermentum* AGR1487.

Although seven tubulin genes had increased expression in response to *L. fermentum* AGR1487, an increase in the abundance of the corresponding tubulin proteins was not detected. This is not surprising given that the transcriptomic analysis was able detect significant expression (higher than background) for greater than 20,000 genes, whereas the proteomic analysis was able to detect significant protein abundance for only 63 proteins due to technical limitations and the sensitivity of the method. However, changes in the abundance of other microtubule-associated proteins in response to *L. fermentum* AGR1487 indicates that observed changes in tubulin gene expression likely resulted in changes to microtubule synthesis.

Increased expression of tubulin genes is also a mechanism used by EPEC that form attaching-and-effacing (A/E) lesions characterised by cytoskeletal alterations, disruption of the brush border cytoskeleton and proliferation of filamentous actin beneath adherent bacteria. EPEC induced A/E lesions also lead to morphological alteration of tight junctions [15], [27]. Our results show that *L. fermentum* AGR1487 cells were required for the negative effect on barrier integrity (bacterial supernatant did not cause a decrease in TEER), suggesting that the physical interaction may be necessary for this effect. Increased adherence of *L. fermentum* AGR1487 to Caco-2 cells (compared to *L. fermentum* AGR1485) is likely to facilitate this cell-to-cell interaction.

In contrast to *L. fermentum* AGR1487, *Lactobacillus plantarum* MB452 has been previously shown to reduce Caco-2 cell expression levels of tubulin genes [22]. *L. plantarum* MB452 is a probiotic bacterium that increases TEER, and its reduction of tubulin turnover is thought to contribute at least in part to this effect by reducing tight junction disassembly. This increase in tight junction integrity is considered beneficial because it strengthens the epithelial barrier which is important for protecting the host from entry of unwanted antigens and pathogens. It appears that different lactobacilli strains can use this mechanism of modifying tubulin gene expression levels to alter intestinal barrier integrity in either a positive (*L. plantarum* MB452) or negative (*L. fermentum* AGR1487) way.

The gene expression results indicate that *L. fermentum* AGR1487 may also decrease tight junction integrity by influencing mitogen-activated protein kinase (MAPK) signalling, however further work is require to confirm this. Caco-2 cells treated with *L. fermentum* AGR1487 had higher expression levels of genes involved in the KEGG *MAPK signaling pathway*, which is known to lead to tight junction disruption [28]. In addition, MAPK is known to directly associate with the microtubule cytoskeleton and there are correlations between MAPK activities and cytoskeletal alterations [29].

As well as indicating possible mechanisms for the negative effect of *L. fermentum* AGR1487 on intestinal barrier integrity, the data also uncovered potential anti-tumour properties of *L. fermentum* AGR1485. Protein expression analysis showed that Caco-2 cells treated with *L. fermentum* AGR1485 had decreased abundance of numerous proteins (histones and others) involved in tumourigenesis. Anti-tumour effects have also been reported with another *L. fermentum* strain; *L. fermentum* RM28 restricted the proliferation of colon cancer cells by 22–29% [30]. Although changes in protein abundance were observed in our study, analysis of cell cycle progression showed no differences in the number of Caco-2 cells in any cell cycle phase between those treated with *L. fermentum* AGR1485 and AGR1487. However, this may be due to the time frame of the treatment (8 hours), which may have been too short to observe differences in cell phases between treatment groups, given that the doubling time of Caco-2 cells is approximately 36 hours [31]. Further research is needed to confirm this potential beneficial anti-tumour effect of *L. fermentum* AGR1485.

The detrimental properties of *L. fermentum* AGR1487 are specific to this particular strain and not typical of all *L. fermentum* strains; *L. fermentum* AGR1485 did not have the same negative effects. *L. fermentum* AGR1487 was isolated from the mouth of a person who was later diagnosed with IBD. IBD is likely due to an unrestrained immune response to commensal bacteria and has been linked to impaired intestinal barrier integrity [32]. Lactobacilli found in human faeces can originate from the mouth [33], [34], indicating that this oral isolate may reside in the intestines of the individual from whom it was isolated. Therefore, it is possible that this *L. fermentum* strain may play a role in disease progression in this particular genetically-susceptible individual. However, based on the current data it cannot be determined whether *L. fermentum* AGR1487 is pathobiontic (detrimental to susceptible individuals only), pathogenic (detrimental to healthy individuals) or symbiotic (not detrimental *in situ*), so further research is needed before conclusions can be drawn about the effect of this strain on human hosts.

In conclusion, this research showed that *L. fermentum* AGR1487 may negatively impact intestinal barrier integrity by increasing the turnover of microtubule proteins triggering tight junction disassembly. Current studies are investigating the effect of *L. fermentum* AGR1487 in mono-associated rats to determine whether the negative effects observed *in vitro* correspond with *in vivo* phenotypic differences. Additionally, the genome sequences of the two *L. fermentum* strains (AGR1485 and AGR1487) are being analysed for differences that may be responsible for the contrasting effects. These findings illustrate that contrasting host cell responses occur in response to bacterial strains of the same species. Given that *L. fermentum* is commonly found in food products, these results imply that food-safe status should be given to individual strains not species and that genome sequence information may be required to confirm strain associated genotypes.

## Supporting Information

Table S1 **Genes that were differentially expressed between Caco-2 cells treated control media and those treated with** ***L. fermentum*** **AGR1485 for 8 hours.**
(XLSX)

Table S2 **Genes that were differentially expressed between Caco-2 cells treated control media and those treated with** ***L. fermentum*** **AGR1487 for 8 hours.**
(XLSX)

Table S3 **Comparison between microarray and qPCR analysis of the gene expression of Caco-2 cells untreated or treated with** ***L. fermentum*** **AGR1485 and** ***L. fermentum*** **AGR1487 for 8 hours.**
(DOCX)

Text S1 **Gene and protein expression methods.**
(DOCX)

## References

[1] Round JL, Mazmanian SK (2009) The gut microbiota shapes intestinal immune responses during health and disease. Nature Reviews: Immunology 9: 313–323. 10.1038/nri2515 PMC4095778 19343057

[2] Chow J, Tang H, Mazmanian SK (2011) Pathobionts of the gastrointestinal microbiota and inflammatory disease. Current Opinion in Immunology 23: 473–480. 21856139 10.1016/j.coi.2011.07.010 PMC3426444

[3] Geier MS, Butler RN, Giffard PM, Howarth GS (2006) *Lactobacillus fermentum* BR11, a potential new probiotic, alleviates symptoms of colitis induced by dextran sulfate sodium (DSS) in rats. International Journal of Food Microbiology 114: 267–274. 17150273 10.1016/j.ijfoodmicro.2006.09.018

[4] Geier MS, Smith CL, Butler RN, Howarth GS (2009) Small-intestinal manifestations of dextran sulfate sodium consumption in rats and assessment of the effects of lactobacillus fermentum BR11. Digestive Diseases and Sciences 54: 1222–1228. 19005763 10.1007/s10620-008-0495-4

[5] Smith CL, Geier MS, Yazbeck R, Torres DM, Butler RN, et al (2008) *Lactobacillus fermentum* BR11 and fructo-oligosaccharide partially reduce jejunal inflammation in a model of intestinal mucositis in rats. Nutrition and Cancer 60: 757–767. 19005975 10.1080/01635580802192841

[6] Mane J, Loren V, Pedrosa E, Ojanguren I, Xaus J, et al (2009) Lactobacillus fermentum CECT 5716 prevents and reverts intestinal damage on TNBS-induced colitis in mice. Inflammatory Bowel Diseases 15: 1155–1163. 19266568 10.1002/ibd.20908

[7] Zoumpopoulou G, Foligne B, Christodoulou K, Grangette C, Pot B, et al (2008) Lactobacillus fermentum ACA-DC 179 displays probiotic potential in vitro and protects against trinitrobenzene sulfonic acid (TNBS)-induced colitis and Salmonella infection in murine models. International Journal of Food Microbiology 121: 18–26. 18077037 10.1016/j.ijfoodmicro.2007.10.013

[8] Olivares M, Díaz-Ropero MP, Sierra S, Lara-Villoslada F, Fonollá J, et al (2007) Oral intake of Lactobacillus fermentum CECT5716 enhances the effects of influenza vaccination. Nutrition 23: 254–260. 17352961 10.1016/j.nut.2007.01.004

[9] Arroyo R, Martín V, Maldonado A, Jiménez E, Fernández L, et al (2010) Treatment of infectious mastitis during lactation: Antibiotics versus oral administration of lactobacilli isolated from breast milk. Clinical Infectious Diseases 50: 1551–1558. 20455694 10.1086/652763

[10] Maldonado J, Cañabate F, Sempere L, Vela F, Sánchez AR, et al (2012) Human milk probiotic lactobacillus fermentum CECT5716 reduces the incidence of gastrointestinal and upper respiratory tract infections in infants. Journal of Pediatric Gastroenterology and Nutrition 54: 55–61. 21873895 10.1097/MPG.0b013e3182333f18

[11] Anderson RC, Cookson AL, Kelly WJ, McNabb WC, Roy NC (2010) *Lactobacillus plantarum* DSM 2648 is a potential probiotic that enhances intestinal barrier integrity. FEMS Microbiology Letters 309: 184–192. 20618863 10.1111/j.1574-6968.2010.02038.x

[12] Ulluwishewa D, Anderson RC, McNabb WC, Moughan PJ, Wells JM, et al (2011) Regulation of tight junction permeability by intestinal bacteria and dietary components. Journal of Nutrition 141: 769–776. 21430248 10.3945/jn.110.135657

[13] Neu J, Sharma R, Young C (2010) Molecular modulation of intestinal epithelial barrier: Contribution of microbiota. Journal of Biomedicine and Biotechnology 2010. 10.1155/2010/305879 PMC2817557 20150966

[14] Ohland CL, MacNaughton WK (2010) Probiotic bacteria and intestinal epithelial barrier function. American Journal of Physiology - Gastrointestinal and Liver Physiology 298: G807–G819. 20299599 10.1152/ajpgi.00243.2009

[15] Guttman JA, Samji FN, Li Y, Vogl AW, Finlay BB (2006) Evidence that tight junctions are disrupted due to intimate bacterial contact and not inflammation during attaching and effacing pathogen infection *in vivo* . Infection and Immunity 74: 6075–6084. 16954399 10.1128/IAI.00721-06 PMC1695516

[16] Karczewski J, Troost FJ, Konings I, Dekker J, Kleerebezem M, et al (2010) Regulation of human epithelial tight junction proteins by Lactobacillus plantarum in vivo and protective effects on the epithelial barrier. American Journal of Physiology: Gastrointestinal and Liver Physiology 298: G851–G859. 20224007 10.1152/ajpgi.00327.2009

[17] Mennigen R, Nolte K, Rijcken EM, Utech M, Loeffler B, et al (2009) Probiotic mixture VSL#3 protects the epithelial barrier by maintaining tight junction protein expression and preventing apoptosis in a murine model of colitis. American Journal of Physiology: Gastrointestinal and Liver Physiology 296: G1140–G1149. 19221015 10.1152/ajpgi.90534.2008

[18] Qin H, Zhang Z, Hang X, Jiang Y (2009) *L. plantarum* prevents enteroinvasive *Escherichia coli*-induced tight junction proteins changes in intestinal epithelial cells. BMC Microbiology 9: 63. 19331693 10.1186/1471-2180-9-63 PMC2674056

[19] Miyauchi E, O'Callaghan J, Butto LF, Hurley G, Melgar S, et al (2012) Mechanism of protection of transepithelial barrier function by Lactobacillus salivarius: strain dependence and attenuation by bacteriocin production. American Journal of Physiology: Gastrointestinal and Liver Physiology 303: G1029–1041. 22961803 10.1152/ajpgi.00003.2012

[20] Hummel S, Veltman K, Cichon C, Sonnenborn U, Schmidt MA (2012) Differential targeting of the E-cadherin/β-catenin complex by gram-positive probiotic lactobacilli improves epithelial barrier function. Applied and Environmental Microbiology 78: 1140–1147. 22179242 10.1128/AEM.06983-11 PMC3272997

[21] Dai C, Zhao DH, Jiang M (2012) VSL#3 probiotics regulate the intestinal epithelial barrier in vivo and in vitro via the p38 and ERK signaling pathways. International Journal of Molecular Medicine 29: 202–208. 22089663 10.3892/ijmm.2011.839

[22] Anderson RC, Cookson AL, Park ZM, McCann M, McNabb WC, et al (2010) *Lactobacillus plantarum* MB452 enhances the function of the intestinal barrier by increasing the expression levels of genes involved in tight junction formation. BMC Microbiology 10: 316. 21143932 10.1186/1471-2180-10-316 PMC3004893

[23] Ormerod MG (2000) Analysis of DNA: General methods. Flow Cytometry: A Practical Approach. Oxford, UK: Oxford University Press. pp. 83–97.

[24] McCann MJ, Gill CI, Linton T, Berrar D, McGlynn H, et al (2008) Enterolactone restricts the proliferation of the LNCaP human prostate cancer cell line in vitro. Molecular Nutrition and Food Research 52: 567–580. 18398867 10.1002/mnfr.200700052

[25] McCann MJ, Rowland IR, Roy NC (2013) Anti-proliferative effects of physiological concentrations of enterolactone in models of prostate tumourigenesis. Molecular Nutrition and Food Research 57: 212–224. 23148045 10.1002/mnfr.201200362

[26] Yap AS, Stevenson BR, Abel KC, Cragoe EJ Jr, Manley SW (1995) Microtubule integrity is necessary for the epithelial barrier function of cultured thyroid cell monolayers. Experimental Cell Research 218: 540–550. 7796888 10.1006/excr.1995.1189

[27] Guttman JA, Li Y, Wickham ME, Deng W, Vogl AW, et al (2006) Attaching and effacing pathogen-induced tight junction disruption *in vivo* . Cellular Microbiology 8: 634–645. 16548889 10.1111/j.1462-5822.2005.00656.x

[28] Costantini TW, Peterson CY, Kroll L, Loomis WH, Eliceiri BP, et al (2009) Role of p38 MAPK in burn-induced intestinal barrier breakdown. Journal of Surgical Research 156: 64–69. 19577248 10.1016/j.jss.2009.03.066 PMC4251589

[29] Reszka AA, Seger R, Diltz CD, Krebs EG, Fischer EH (1995) Association of mitogen-activated protein kinase with the microtubule cytoskeleton. Proceedings of the National Academy of Sciences of the United States of America 92: 8881–8885. 7568036 10.1073/pnas.92.19.8881 PMC41071

[30] Thirabunyanon M, Boonprasom P, Niamsup P (2009) Probiotic potential of lactic acid bacteria isolated from fermented dairy milks on antiproliferation of colon cancer cells. Biotechnology Letters 31: 571–576. 19116692 10.1007/s10529-008-9902-3

[31] Briske-Anderson MJ, Finley JW, Newman SM (1997) The influence of culture time and passage number on the morphological and physiological development of Caco-2 cells. Proceedings of the Society for Experimental Biology and Medicine 214: 248–257. 9083258 10.3181/00379727-214-44093

[32] Bruewer M, Samarin S, Nusrat A (2006) Inflammatory bowel disease and the apical junctional complex. Annals of the New York Academy of Sciences 1072: 242–252. 17057204 10.1196/annals.1326.017

[33] Dal Bello F, Hertel C (2006) Oral cavity as natural reservoir for intestinal lactobacilli. Systematic and Applied Microbiology 29: 69–76. 16423658 10.1016/j.syapm.2005.07.002

[34] Maukonen J, Matto J, Suihko ML, Saarela M (2008) Intra-individual diversity and similarity of salivary and faecal microbiota. Journal of Medical Microbiology 57: 1560–1568. 19018030 10.1099/jmm.0.47352-0

