# Supplementary material for: Human Oral Isolate Lactobacillus fermentum AGR1487 Reduces Intestinal Barrier Integrity by Increasing the Turnover of Microtubules in Caco-2 Cells
Source: PLoS One. 2013 Nov 14;8(11):e78774. doi: 10.1371/journal.pone.0078774 (PMC3828418; doi:10.1371/journal.pone.0078774)
Supplement: Table S3 — Comparison between microarray and qPCR analysis of the gene expression of Caco-2 cells untreated or treated with L. fermentum AGR1485 and L. fermentum AGR1487 for 8 hours. (DOCX) [file pone.0078774.s003.docx]

**Table S3.** **Comparison between microarray and qPCR analysis of the gene expression of Caco-2 cells untreated or treated with *L. fermentum* AGR1485 and *L. fermentum* AGR1487 for 8 hours.**

| **Gene** | **Full gene name** | **TaqMan ID** | **AGR1485 vs Control** | | **AGR1487 vs Control** | | **AGR1487 vs AGR1485** | |
| --- | --- | --- | --- | --- | --- | --- | --- | --- |
|  |  |  | **Microarray fold change** | **qRT-PCR**  **fold change** | **Microarray fold change** | **qRT-PCR**  **fold change** | **Microarray fold change** | **qRT-PCR**  **fold change** |
| FOS | FBJ murine osteosarcoma viral oncogene homolog | Hs00170630_m1 | 1.3^NS^ | 2.28^2^ | 3.14^1^ | 13.38^2^ | 2.43^1^ | 5.87^2^ |
| GCNT3 | glucosaminyl (N-acetyl) transferase 3, mucin type | Hs00953355_m1 | -2.00^NS^ | 4.32^2^ | 1.69^1^ | 9.42^2^ | 2.02^NS^ | 2.18^2^ |
| SLC16A3 | solute carrier family 16, member 3 (monocarboxylic acid transporter 4) | Hs00358829_m1 | 1.80^NS^ | -1.18^NS^ | 1.12^NS^ | 1.30^NS^ | 2.11^1^ | 1.53^2^ |
| CXCL12 | chemokine (C-X-C motif) ligand 12 | Hs00171022_m1 | 1.06^NS^ | -2.01^2^ | 1.03^NS^ | N/A | 1.01^NS^ | 3.45^2^ |
| EDN1 | endothelin 1 | Hs00174961_m1 | 1.80^NS^ | 3.64^2^ | 3.30^1^ | 5.40^2^ | 1.30^NS^ | 1.46^2^ |
| RGS16 | regulator of G-protein signaling 16 | Hs00892674_m1 | 1.54^NS^ | 2.97^2^ | 2.60^1^ | 7.34^2^ | 1.68^NS^ | 2.46^2^ |
| PLK3 | polo-like kinase 3 | Hs00177725_m1 | 2.57^NS^ | 2.35^2^ | 5.17^1^ | 7.63^2^ | 2.01^1^ | 3.24^2^ |
| FGF19 | fibroblast growth factor 19 | Hs00192780_m1 | 1.45^NS^ | 1.51^2^ | 1.97^1^ | 6.01^2^ | 1.43^NS^ | 4.00^2^ |
| CCNA2 | cyclin A2 | Hs00996788_m1 | -1.03^NS^ | -1.59^NS^ | -1.20^1^ | -1.17^NS^ | 1.10^NS^ | 1.38^NS^ |
| SLC26A1 | solute carrier family 26 (sulfate transporter), member 1 | Hs00222784_m1 | -1.01^NS^ | 1.18^NS^ | -1.04^NS^ | 2.78^2^ | 1.01^NS^ | 2.37^2^ |

^1^ FDR < 0.05, differentially expressed between treatment groups

^2^ P < 0.05, differentially expressed between treatment groups

^NS^ Not significantly differentially expressed between treatment groups

N/A: data not available
