# Supplementary material for: Human Oral Isolate Lactobacillus fermentum AGR1487 Reduces Intestinal Barrier Integrity by Increasing the Turnover of Microtubules in Caco-2 Cells
Source: PLoS One. 2013 Nov 14;8(11):e78774. doi: 10.1371/journal.pone.0078774 (PMC3828418; doi:10.1371/journal.pone.0078774)
Supplement: Text S1 — Gene and protein expression methods. (DOCX) [file pone.0078774.s004.docx]

**Supplementary Text S1: Gene and protein expression methods**

**Whole genome expression analysis**

The total RNA was extracted from the Caco-2 cells using TRIzol (Invitrogen, Auckland, New Zealand) according to the manufacturer’s instructions. The RNA was precipitated by the addition of 1 µL glycogen (5 mg/mL), 0.1 volumes 3 M sodium acetate and 2.5 volumes 100% ethanol and incubated at -85°C for 30 minutes prior to centrifugation (12000 x *g* for 30 minutes). The pellet was washed with 100% ethanol and recollected (12000 x *g* for 30 minutes) twice. After drying, RNA was resuspended in 50 µL RNase-free water. This ensured that all contaminating salts were removed from the sample prior to further purification. The RNA quantity and purity was determined using a Nanodrop ND-1000 spectrophotometer (Nanodrop Technologies), and all samples had an OD_260nm_/OD_230nm_ ratio in the range of 1.8-2.2. The extracted RNA was purified using RNeasy mini columns (QIAGEN) and RNA quality was assessed using an RNA 6000 NanoLabChip kit with the Agilent 2100 Bioanalyzer (Agilent Technologies, Palo Alto, CA, USA). All samples had an OD_260nm_/OD_280nm_ ratio greater than 2.0, a Bioanalyzer 28s/18s peak ratio greater than 1.5, and a RNA integrity number (RIN) greater than 8.

The RNA samples were labelled using the Agilent Low RNA Input Linear Amplification Kit PLUS (5183-3523). The total RNA (500 ng) was amplified and reverse transcribed to cDNA using T7-polymerase. The cDNA was labelled with either cyanine3 or cyanine5 labelled CTP dye (Perkin-Elmer/NEN Life Sciences). The amplification and dye incorporation was analysed using a Nanodrop ND-1000 spectrophotometer. The amplified and fluorescently labelled cRNA was hybridised in a loop design using the Agilent Gene Expression Hybridisation Kit (5188-5242-A). 825 ng of each labelled cRNA (Cy 3 & Cy 5) were combined in a hybridisation mix, which was subjected to a fragmentation reaction at 60°C for 30 minutes. Following this, 100 µl of the hybridisation mix was loaded onto a 4 x 44k gasket slide and sandwiched with an Agilent 4 x 44k human microarray, held securely in place by a hybridisation chamber. Following hybridisation the chambers were dissembled and the microarray slides were washed in Agilent Gene Expression Wash Buffer 1 (5188-5325), Gene Expression Wash Buffer 2 (5188-5326), acetonitrile and Stabilisation and Drying Solution (5185-5979). The slides were scanned using a GenePix Professional 4200A scanner (Molecular Devices, Sunnyvale, CA, USA). Spot identification and quantification was completed with GenePix 6.0 software (Molecular Devices). The images were also manually checked and any poor quality spots were removed before statistical analysis.

Microarray data were analysed in R (version 2.15.1) using the limma package [[1](#_ENREF_1)]. Intensity ratios for all microarray features were normalised using a Loess smoothing algorithm without background correction. For each spot, a modified t-test was calculated using an empirical Bayes approach [[2](#_ENREF_2)]. Genes with a fold change greater than 1.5 and a multiple testing adjusted false discovery rate (FDR) less than 0.05 were considered differentially expressed. Differentially expressed genes were analysed for over-representation among KEGG pathways using a hypergeometric test in R using the GOstats package [[3](#_ENREF_3)]. Hierarchical clustering of average gene expression among KEGG pathways was generated in R. Class discrimination of gene expression profiles by treatment groups was completed using PLS-DA from the mixOmics package for R [[4](#_ENREF_4)]. PLS-DA is a dimension reduction approach combined with a regression mode that, unlike Principle Component Analysis (PCA; an unsupervised method), finds components that are also relevant to the treatment. Correspondence analysis (CA) of *Gap* *Junction* gene expression profiles was done using the MADE4 package for R [[5](#_ENREF_5)]. CA is a dimension reduction method similar to PCA, but also allows the simultaneous projection of the variables (genes) that are responsible for the separation of samples.

**Validation of gene expression data**

To validate the microarray data, the expression levels of ten selected differentially expressed genes in the Caco-2 cell RNA samples were quantified with TaqMan probe chemistry-based real-time PCR (qPCR) (Supplementary Table 3). The genes chosen had a range of expression patterns across the microarray analysis and are involved in a range of biological processes. All reagents were obtained from Applied Biosystems (Life Technologies) unless otherwise stated. For each sample, 500 ng of total RNA was reverse transcribed into cDNA using a high capacity RNA-to-cDNA kit as follows; 10 µL of 2x RT buffer, 1.0 µL of 20x RT enzyme mix, and 9.0 µL of total RNA in nuclease-free water. A thermal cycle of 37°C for 1 hour and 95°C for 5 minutes was used for these reactions and the cDNA stored at -20°C. The quantification of the expression levels of the ten selected genes relative to three reference genes was completed on the RotorGene 6000 qPCR instrument (Qiagen, NZ). The reference genes were SDHA (succinate dehydrogenase complex, subunit A, flavoprotein (Fp); TaqMan ID: Hs00188166_m1), YWHAZ (tyrosine 3-monooxygenase/tryptophan 5-monooxygenase activation protein, zeta polypeptide; TaqMan ID: Hs03044281_g1), and GAPDH (glyceraldehyde-3-phosphate dehydrogenase; TaqMan ID: Hs03929097_g1). Each qPCR (no-template controls, untreated controls and samples) was prepared as triplicate 20 µL reactions composed of: 10.0 µL of 2x master mix, 1.0 µL of 20x gene expression assay, and 9.0 µL of a 1 in 10 dilution of cDNA. The thermal profile used was; 50°C for 2 minutes, 95°C for 10 minutes, and 40 cycles of 95°C for 15 seconds and 60°C for 60 seconds. The expression levels of these genes were analysed using the relative expression software tool (REST, version 2.0.13). Two independent assay runs were completed. Correlation between microarray and qPCR results was tested by Spearman rho correlation analysis in R.

**Protein expression analysis**

The protein samples isolated from the Caco-2 samples during the TRIzol extraction (co-current with RNA extraction) were centrifuged and the pellets were dissolved through sonication in 100 µL 0.5 M triethylammonium bicarbonate (TEAB) buffer with 2% sodium deoxycholate. A protein assay was completed using the 2D Quant kit (GE Healthcare). Sample amounts were equalised to 33 µg and vacuum-centrifuged to dryness. Samples that showed little or no presence of protein were also vacuum-centrifuged to dryness and processed identically. Dehydrated proteins were reconstituted in 20 µL 0.5 M TEAB. Reduction was achieved by adding 1 µL of 200 mM dithiothreitol in 0.5 M TEAB and incubating at 56°C for 45 minutes. The samples were then alkylated by adding 1 µL of 600 mM iodoacetamide for 30 minutes in the dark. The proteins were digested by adding 5 µg of sequencing grade porcine trypsin (Promega) and incubating overnight at 37°C. Each digested sample was isobarically labelled for 2 hours at room temperature by adding to each the contents of one vial of iTRAQ 8plex reagent (ABSciex). Specifically, 24 samples were combined in four iTRAQ experimental sets. Channels 113 to 118 were allocated to the 24 samples while channel 119 was a pool of all samples allowing comparison and relative quantitation across all samples in the four iTRAQ experiments. The labelling reaction was stopped by the addition of 100 µL of LCMS-grade water and incubating at room temperature for 30 minutes. The samples in each set were pooled and vacuum centrifuged to dryness.

Prior to analysis, dried samples were dissolved in 50 µL of 0.1% formic acid and 2% acetonitrile in LCMS-grade water. LC-MS/MS was completed on a Dionex nano RSLC (Dionex) coupled to a Bruker maXis impact mass spectrometer equipped with a CaptiveSpray source (Bruker Daltonik, Bremen, Germany). For each sample, 2 µL was loaded on an Acclaim PepMap RSLC nano trap (Dionex, 75 μm x 2 cm, C18, 3 μm particles, 100 Å pore size). The trap column was then switched in line with the analytical column (Dionex Acclaim PepMap RSLC, 75 μm x 25 cm C18, 2 μm particles, 100 Å pore size). The column oven temperature was 40°C. Elution was with a gradient from 4% to 50% of solvent B in 90 minutes at a flow rate of 300 nL/minute. Solvent A was LCMS-grade water with 0.1% formic acid (FA); solvent B was LCMS-grade acetonitrile with 0.1% FA. Samples were measured in auto MS/MS mode, with a mass range of m/z 50-2200. After an MS spectrum was taken, the 20 most intense ions in that spectrum were selected for MS/MS (fragmentation). After this MS/MS cycle, a new MS spectrum was acquired. The instrument was cycled this way between MS and MS/MS for the duration of the chromatographic run. Acquisition speed was 2 Hz in MS and 10 or 5 Hz in MS/MS mode depending on precursor intensity. Precursors were selected in the m/z 400-1400 range, with charge states 2-5 (singly charge ions were excluded). Active exclusion was activated after 1 spectrum for 0.3 minutes.

Peak list files (mgf format) were generated using DataAnalysis (Bruker) and were submitted to an in-house Mascot server (v2.2.0.6) (Matrix Science, UK). The following search parameters were used: Taxonomy Homo sapiens; Enzyme semitrypsin; Cysteine modification carbamidomethyl; MS tolerance 15 ppm; MS/MS tolerance 0.05 Da; 1 missed cleavage; instrument specificity ESI-QUAD-TOF. Mascot iTRAQ parameters included fixed iTRAQ8plex (N-term, K) and variable iTRAQ8plex (Y), with reporter ions defined as appropriate for the experiment. Data analysis was completed with the aid of the IsobariQ v1.3.1 software [[6](#_ENREF_6)]. For each iTRAQ experiment, the corresponding Mascot result file was loaded in IsobariQ, the reporter ratios defined with the total pool channel as common denominator, and a reporter ion correction matrix applied (iTRAQ 8 plex with phenylalanine). Peptides with a score below 15, and proteins with fewer than 2 peptides were discarded. Quantitation was completed in IsobariQ using normalisation based on division by channel sum. The quantitation results from the four iTRAQ assays were combined for each protein, with the total pool channel facilitating comparison between iTRAQ experiments.

Differentially expressed proteins were determined using the non-parametric Kruskal-Wallis one way analysis of variance method in R. PLS-DA of protein expression profiles was performed using the mixOmics package in R. Procrustes rotation analysis of gene and protein PLS-DA projections was completed using the vegan package in R [[7](#_ENREF_7)]. Procrustes rotation analysis is a method for rotating and scaling points from one ordination, such as projections from a PLS-DA plot in this case, to be as close as possible to points from another ordination, while maintaining the relative distances between points within each ordination.

**References**

[1] Smyth, G., in: Gentleman, R., Carey, V., Dudoit, S., Irizarry, R., Huber, W. (Eds.), *Bioinformatics and Computational Biology Solutions using R and Bioconductor*, Springer, New York 2005, pp. 397–420

[2] Smyth, G. K., Linear Models and Empirical Bayes Methods for Assessing Differential Expression in Microarray Experiments. *Statistical Applications in Genetics and Molecular Biology* 2004, *3*, Article 3.

[3] Falcon, S., Gentleman, R., Using GOstats to test gene lists for GO term association. *Bioinformatics* 2007, *23*, 257-258.

[4] Le Cao, K. A., Gonzalez, I., Dejean, S., integrOmics: an R package to unravel relationships between two omics datasets. *Bioinformatics* 2009, *25*, 2855-2856.

[5] Culhane, A. C., Thioulouse, J., Perriere, G., Higgins, D. G., MADE4: an R package for multivariate analysis of gene expression data. *Bioinformatics* 2005, *21*, 2789-2790.

[6] Arntzen, M. Ø., Koehler, C. J., Barsnes, H., Berven, F. S.*, et al.*, IsobariQ: Software for isobaric quantitative proteomics using IPTL, iTRAQ, and TMT. *Journal of Proteome Research* 2011, *10*, 913-920.

[7] Oksanen, J., Blanchet, F. G., Kindt, R., Legendre, P.*, et al.*, vegan: Community Ecology Package. R package version 2.0-4. 2012, [*http://CRAN.R-project.org/package=vegan*](http://CRAN.R-project.org/package=vegan).
